# A cross-sectional survey analysis of patient and family knowledge, confidence, and perceived barriers to reporting patient deterioration

**DOI:** 10.1371/journal.pone.0319546

**Published:** 2025-03-11

**Authors:** Lisa Thiele, Arthas Flabouris, Campbell Thompson

**Affiliations:** 1 The University of Adelaide, Faculty of Health and Medical Sciences, Adelaide Medical School, Adelaide, South Australia, Australia; 2 Intensive Care Unit, Royal Adelaide Hospital, Adelaide, South Australia, Australia; 3 General Medicine Service, Royal Adelaide Hospital, Adelaide, South Australia, Australia; Universiteit Antwerpen, BELGIUM

## Abstract

**Background:**

The knowledge, confidence, and skills of healthcare consumers to identify acute clinical deterioration and appropriately escalate concerns remain largely undetermined. This gap is despite the widespread international introduction of consumer escalation systems intended to provide patients and family an avenue to escalate their concerns if worried about deterioration in their own or relative’s condition during a hospital stay.

**Aim:**

To explore patient and family knowledge of acute clinical deterioration, and their confidence and perceived barriers to escalating their concerns.

**Design:**

Cross-sectional, in-person, consumer surveys across an Australian acute adult hospital. The study specific survey tool was developed through a multistage process with healthcare consumer input during creation and testing.

**Methods:**

Questions explored healthcare consumer knowledge, confidence, and perceived barriers in association with acute clinical deterioration, recognising deterioration, and escalating concerns. Descriptive and inferential analysis was completed, and knowledge, confidence, and barrier scores established. Association between scores and consumer type, gender, age, education level, prior experience with clinical deterioration or rapid response team review, and hospitalisation history in the last 12 months were assessed using multivariable linear regression.

**Results:**

133 surveys were completed. Knowledge scores varied across respondents. Awareness of the local consumer escalation system was low. A positive association was identified between knowledge and confidence that diminished with increasing barrier scores. A strong negative correlation was present between barriers and confidence. No significant difference existed in knowledge, confidence, or barrier scores based on consumer type, gender, education level, previous experience with deterioration or rapid response team review, or hospitalisation history.

**Conclusions:**

Limitations in patient and family knowledge may impede consumer escalation system success. Increasing knowledge may enhance patient and family confidence to identify deterioration and escalate concerns. However, barriers to consumer escalation decrease this potential. Interventions to increase consumer knowledge should therefore be accompanied by strategies to minimise barriers.

## Introduction

Clinician activated rapid response systems (RRS) are designed to achieve early detection and response to acute clinical deterioration [1]. However, significant adverse outcomes have occurred in cases where patient and family concerns of clinical deterioration have received insufficient response [2]. Such adverse events have driven the introduction of consumer (that is patient and family or carer) escalation systems (CES) [2–4]. CES are intended to provide an avenue for patients and families to escalate their concerns about a worsening in their own or loved one’s condition during a hospital stay [3–5]

The importance of introducing systems to allow patients and families to escalate concerns of clinical deterioration has been recognised internationally. This has seen CES become a health service requirement within regions such as the United States of America [6], United Kingdom [7], Australia [5], and New Zealand [8]. CES exist under a variety of names and models [3,9]. Differences are present in activation criteria, with some systems being solely focused upon clinical deterioration in comparison to general concern in others [3,9]. Differences are also present in the activation mechanisms and escalation pathway available to the patient and family. To this respect, a system may be considered as indirect, where the patient or family can only escalate their concerns incrementally through their primary care team or to a dedicated consumer escalation team or representative who then assess whether a Rapid Response Team (RRT) is required [9,10]. Alternatively, direct activation allows the patient or family to bypass such steps and independently activate a RRT [9,10]. A third hybrid model also exists in which the patient or family can escalate their concerns through three pathways, that being through their primary care team, by requesting staff call a RRT on their behalf, or via direct patient/family activation of the RRT [11]. Consensus of an ideal model is yet to be determined [9]

Engaging with and empowering patients and families is recognised as one of the most powerful means of enhancing patient safety [12]. CES should support this intention. However, despite emerging over 15 years ago, robust data to demonstrate a positive effect of CES on patient outcomes remains limited [3,9]. Potential barriers have been identified to effective system functioning [9,11]. CES are a multifaceted intervention that challenge traditional hospital hierarchies, culture, and practice [11,13], with potential to be impacted upon by a complicated interplay of factors [4]. Our previous research explored the perceptions of staff [14]. Another essential consideration relates to the capability of patients and families to participate in the consumer escalation process [13]. This capability relates to patient and family awareness of deterioration and of the escalation system and their ability to recognise deterioration and successfully voice and escalate concerns [13]. Gaining a better understanding of this capability as it relates to patient and family knowledge, confidence, and barriers to escalation is crucial if CES are to achieve their full intention.

### Aim

To examine patient and family knowledge, confidence, perceived barriers, and facilitators, in identifying acute clinical deterioration and escalating their concerns.

## Materials and methods

### Design

Cross-sectional in-person surveys were conducted with patients, family or carers between the 5^th^ of March and 25^th^ of November 2021. Consenting participants completed the survey on one occasion with no follow up requirements. Surveys were conducted within the context of a larger mixed-methods project incorporating patient and family interviews post RRT review and staff surveys exploring clinician perceptions of CES [14]. The EQUATOR Network Checklist for Reporting Of Survey Studies (CROSS) [15] was adopted and guided study development, conduction, and reporting.

### Study setting

The study was conducted within an 800-bed quaternary, Australian adult public teaching hospital, with an established clinician activated RRS. Study participants were sought across medical, surgical, and speciality wards. Examples of speciality wards included cardiology, haematology/oncology, gastroenterology, neurology, renal, spinal, and thoracic services. The Emergency Department and Intensive Care Unit were excluded as they operated under different escalation processes. The Mental Health Unit was also excluded as the study was focused upon physiological deterioration. The hospital does not provide inpatient paediatric or maternity services.

**Fig 1:**
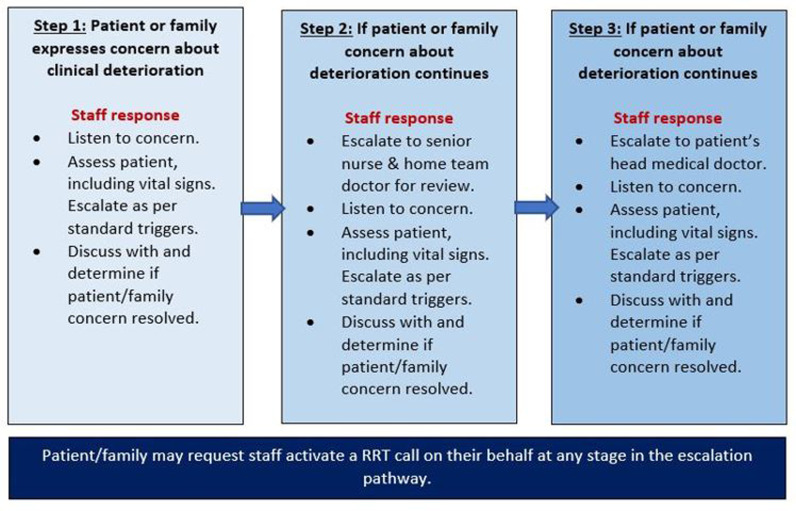
Local consumer escalation process (adapted from Local Health Network (CALHN) procedure 2019) [16].

A CES was introduced across the hospital in 2019. This system represented an indirect model, where patients and family were encouraged to escalate concerns using a tiered response through their primary care team [16]. Patients and families could also request staff activate a RRT call on their behalf. As per hospital protocol, staff were required to comply with this request, even if no other RRT activation triggers were present. Fig 1 represents the local escalation pathway [16]. Consumer facing materials outlined the escalation steps and options. At the time of data collection, patients and families did not have a means to directly activate the RRT without requesting staff do so on their behalf.

Consumer information that outlined the system’s purpose and escalation steps was available in the form of brochures, posters, digital displays, and online resources. A hospital procedure document [16], an on-line learning package, and in-services were available to staff. Staff material outlined system purpose and that clinicians were responsible for ensuring that patients and their family members/carers were educated about the CES at regular intervals. For example, on admission, during clinical handovers or rounds, and at transfer of care between inpatient locations [16]. Information also included the CES steps and required staff actions if a patient or family expressed concern.

### Survey tool development

**Fig 2:**
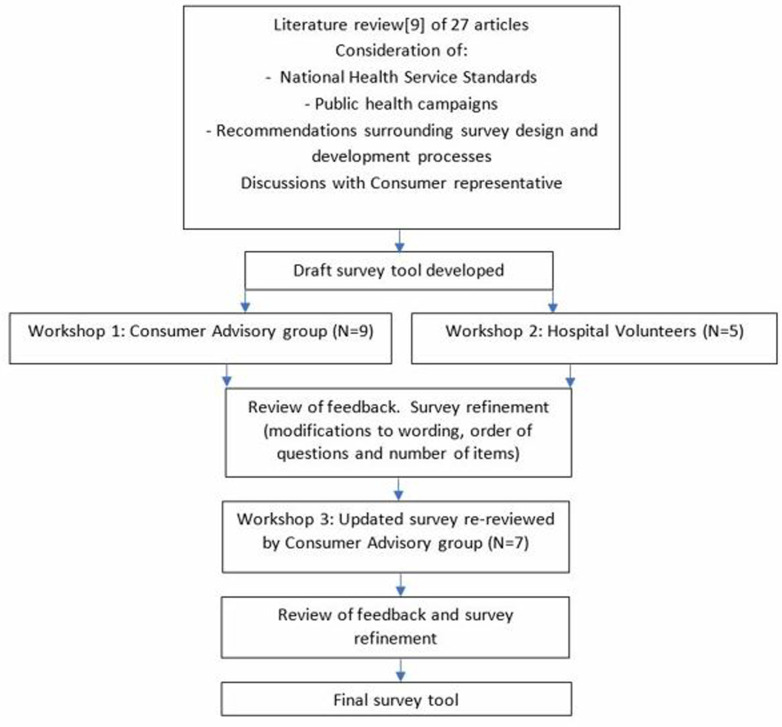
Survey tool development process.

Owing to the absence of a pre-existing tool specific to the study’s aims, a new instrument was created through a multi-stage process (Fig 2). A comprehensive literature review [9] was completed to evaluate the current evidence and inform members of the research team who jointly developed a draft survey tool. Consumer input was sought through participation in a series of workshops. The workshops focused on achieving survey question appropriateness, clarity, and face validity, as was assessed through group discussion and consensus. Prior to commencing data collection, the survey tool was tested with a separate group of 14 consumer representatives (seven males and seven females, age brackets ranging from 30 to 70 years). Eight volunteers tested an online version of the survey and six completed the survey in-person. The latter method was found to overcome difficulties associated with computer literacy/usability reported by four volunteers during the testing process. Testing the survey in the in-person format additionally allowed the researcher who was completing data collection to gain experience in survey delivery.

Survey questions primarily focused on knowledge, confidence, and perceived barriers and facilitators related to identifying acute clinical deterioration and escalating concerns. Knowledge and confidence were identified as critical topics for survey inclusion, as both relate to patient enablement and empowerment, which are the precursors to involvement and engagement in care [17]. Engagement has in turn been associated with improved patient outcomes [18]. Furthermore, links have previously been recognised between health-related confidence and patient knowledge, ability to access care, shared decision making, and patient capability [19], all of which are relevant to CES. The need to evaluate knowledge and confidence have also been recognised in prior literature associated with CES [9,20]. The inclusion of perceived barriers (and conversely facilitators) as the third focus area was grounded on prior research highlighting the importance of considering barriers to CES functioning [9,11]. Finally, knowledge, confidence and barriers were felt to be key considerations associated with health literacy.

Within the study information sheets and at the commencement of the survey, participants were provided with a definition of acute clinical deterioration of ‘*when the medical condition of a patient in hospital suddenly or unexpectedly worsens’.*

In exploring knowledge, the survey included a list of signs and symptoms from existing public health campaigns for sepsis [21], stroke [22], acute coronary syndrome [23], and heart failure [24]. Items not associated with acute clinical deterioration were also included to assist in identifying response bias. The wording used within the list was that agreed upon within the consumer workshops. A four-point Likert scale was used to explore patient and family perceptions, and knowledge, of the signs and symptoms that they associated with acute clinical deterioration. Knowledge of vital signs was assessed by asking participants what they considered to be normal for conscious state, pulse rate, respiratory rate, and systolic blood pressure after being presented with either multiple choice (conscious state) or scale (remaining vital sign) options. Correct responses were conscious state: ‘wakes easily when spoken to and stays awake’, pulse rate: 60 to 100/minute, respiratory rate: 12 to 20/minute, and systolic blood pressure: > 100 and < 130mmHg. Additional survey questions examined patient and family awareness of the local CES and considered the steps involved in escalating concerns within this system.

Using a five-point Likert scale, seven survey questions explored participant confidence to identify an acute change in their own or family member’s medical condition, confidence in their own medical knowledge, and confidence to escalate concerns. Three of the questions related to escalating concerns focused on the steps in the local CES pathway. Another 11 questions explored perceived barriers and facilitators to recognising and escalating concerns of acute deterioration. Based on literature review findings [9] and the outcomes of consumer workshops, questions primarily explored patient and family perceived right to raise concerns, perceived knowledge of oneself or relative’s baseline status, preferred role in care (that being active versus passive), and the potential impact that hospital staff can have on facilitating or preventing CES.

Respondent characteristics, hospitalisation history, and prior experience with clinical deterioration (either self-reported or based on a prior RRT review) were collected through yes/no and multiple-choice questions. For questions about history and prior experience, surveyed family members reported upon the patient who they were accompanying.

Two versions of the survey were developed, with wording specific to either patients or family members/carers, respectively (S1 File).

### Inclusion and exclusion criteria

Surveys were open to adult inpatients and accompanying family members. ‘Family’ was defined as an individual who a patient would consider to be of significance and who would have a unique insight into the patient’s normal status. Prior experience with clinical deterioration or a RRT review was not a specific inclusion criterion for survey participation. However, as the surveys were completed as part of a larger mixed methods study, a subgroup of the participants who additionally completed an interview had experienced a prior RRT review.

As the study was focused on the adult population, individuals aged less than 18 years were excluded. Other exclusion criteria included inability to provide informed consent or verbalise responses, medical instability (as assessed by abnormal vital signs), the presence of a detention order, and patients excluded by the admitting medical team or senior ward nursing staff. Finally, non-English-speaking individuals were excluded as, given the complexity of the survey and available study resources, it would not have been possible to access translation support to ensure informed consent or understanding of research questions.

### Sampling

Based on the aim of the study and the available resources, the main sampling method adopted was that of convenience, with participants enrolled based on their accessibility and availability [25]. Participants were identified by a member of the research team (LT) visiting wards to screen for eligible individuals. Where the screening process identified a number of eligible individuals that exceeded the capacity of daily study resources to approach, to ensure a fair process [26], a random number generating tool was used to select bed numbers and therefore potential participants. Where only a limited number of potential participants existed, all available were approached.

In addition to the convenience sampling method that was undertaken, as the surveys were completed in the context of a larger mixed methods study that also included qualitative interviews, purposeful sampling was adopted in a subgroup of participants who were identified based on having had a recent RRT review. This group of 22 individuals completed both the survey that formed part of this study, and an interview associated with the qualitative investigation.

The sample size was determined by that which could be obtained in the data collection period with the available resources. This was impacted upon by intermittent visitor restrictions associated with the COVID 19 pandemic during the data collection period. Consideration was also given to the aim of the study and the nature of the previous literature associated with CES.

### Data collection

The Research Electronic Data Capture (REDCap) tool and platform hosted at our institution was used for data collection and initial management [27].

Potential participants were approached and invited to participate in the study by a single member of the research team (LT). To minimise the risk of dependent or unequal relationship impacting upon an individual’s decision, the researcher was not directly involved in the patient’s daily care. No reimbursement was offered for study participation.

Participants were provided with a study information sheet, advised that participation was voluntary, that they could choose not to answer specific questions, and that they could withdraw, at any stage, prior to submitting their final survey responses. Written informed consent was obtained from all participants upon consent forms and witnessed by the study investigator. All surveys were completed in-person within a quiet location in the hospital. Participant responses were entered by the member of the research team conducting surveys. To minimise errors in data entry, responses were documented directly into an electronic version of the survey on a tablet device after each question.

All collected data was stored on the Hospital’s username and password protected computer network with access restricted to members of the research team. All participant identifiers were removed from collected data prior to analysis.

### Data analysis

Data analysis was completed using IBM SPSS Statistics (Version 27) and Strata statistical software program (Version 17) using descriptive and inferential approaches.

The Chi-square, Fisher Exact test, and Fisher-Freemand-Halton Test were used as appropriate to examine differences in the distribution of demographics between patient and family respondents, and in their responses to individual Likert items. Analyses included all available responses for each individual question.

To explore and summarise patient and family knowledge of signs and symptoms and vital signs, confidence to identify deterioration and escalate concerns, and perceived barriers and facilitators, related questions were clustered during analysis.

A knowledge score was derived by awarding one point for each sign or symptom correctly identified as indicative of acute clinical deterioration (that is, the participant provided an “absolutely” response when asked if they considered the sign or symptom to be an indicator of acute clinical deterioration). For the three items not associated with acute deterioration, one point was instead awarded for a “definitely not” response. A further one point was also awarded for each vital sign response falling within the pre-determined ranges. Points were summed as a total knowledge score ranging from 0 to 25, with 25 reflecting the highest level of knowledge.

Confidence and barrier/facilitator scores were examined through Likert scales. Responses to the individual Likert items were ranked and assigned a numeric value from one to five. The values from related survey items were then summed to produce summary Likert scale-type scores. The resultant Likert scales (unlike individual Likert items) can be considered as interval in nature, thus permitting parametric analyses [28]. Confidence scores were derived by summing the responses for seven related survey items, such that higher scores reflect higher levels of confidence (total range 7-35). In examining barriers and facilitators, a reverse scoring system was applied such that for each item, the response associated with the greatest barrier was scored five and the response with the greatest facilitator scored one (total range 11-55). Consequently, the higher the score, the greater the perceived barrier present. This summary score is hereafter referred to as the barrier score. Confidence and barrier scoring systems only included those who provided responses to all associated questions. Full details of each scoring system are provided in S2 File.

Overall knowledge of signs and symptoms, confidence, and barrier scores were compared for patients and family through descriptive measures and an independent samples t-test. Associations between each of knowledge, confidence, and barrier scores and the pre-identified factors of consumer type (patient or family), gender, age category (≤40 years, 41-60 years, 61-80 years, > 80 years), highest level of education, prior experience with clinical deterioration or RRT review, and number of hospital admissions and length of stay were assessed using multivariable linear regression. The relationships between knowledge, confidence, and barrier scores were described using Pearson’s correlations and examined further using the multivariable linear regression. For continuously measured predictor variables, a linear relationship with the outcome was assumed. Model adequacy for all regression models was assessed by visual inspection of the residuals for normality and homoscedasticity. Furthermore, to explore whether relationships between knowledge and confidence differed according to perceived barriers, interaction terms were included as appropriate. Finally, three separate independent samples median tests were used to examine whether scores differed according to respondent awareness of the local CES.

### Ethical considerations

Local Human Research Ethics Committee and Governance approvals were obtained (Central Adelaide Local Health Network HREC Reference Number: 13231; Governance Reference Number: P2275). This approval was accepted by The University of Adelaide Human Research Ethics Committee (Reference Number: 34660).

## Results

### Sample characteristics

237 individuals were approached. 74 declined. From 24 individuals who requested further time to consider their decision, 16 were lost to discharge and eight were excluded secondary to alteration in their clinical condition. Three participants demonstrated sufficient English to complete consent processes; however, were excluded after survey commencement as the study investigator assessed that limitations with English proficiency were impacting upon their ability to accurately comprehend all survey questions. Surveys were also discontinued with two individuals in whom hearing limitation impacted on their ability to comprehend the extended nature of survey items. Finally, one participant who had previously completed the survey was re-approached during a second later hospital admission and was therefore excluded on this occasion. Consequently, 133 surveys were completed (90 inpatients and 43 family members) giving a response rate of 56.1% (133/237). 22 of the participants were identified through purposeful sampling as part of qualitative interviews completed within the wider mixed method study. Respondent characteristics are summarised in Table 1.

**Table 1:** Respondent characteristics.

Characteristic	Consumer Patient (N = 90)	Family (N = 43)	P value
**Gender**			<0.001
Female	46/90 (51.1%)	36/43 (83.7%)	
**Age group**			0.013
< 30 years	7/90 (7.8%)	3/43 (7.0%)	
31-40 years	1/90 (1.1%)	2/43 (4.7%)	
41-50 years	7/90 (7.8%)	7/43 (16.3%)	
51-60 years	18/90 (20.0%)	14/43 (32.6%)	
61-70 years	22/90 (24.4%)	13/43 (30.2%)	
71-80 years	24/90 (26.7%)	3/43 (7.0%)	
> 80 years	11/90 (12.2%)	1/43 (2.3%)	
**Highest level of education**			0.491
Primary school	4/90 (4.4%)	1/43 (2.3%)	
Secondary school	34/90 (37.8%)	13/43 (30.2%)	
Vocational/apprenticeship	17/90 (18.9%)	6/43 (14.0%)	
University	35/90 (38.9%)	23/43 (53.5%)	
**Area of work**			0.024
Agriculture	3/90 (3.3%)	1/43 (2.3%)	
Business/Trade/Office	11/90 (12.2%)	4/43 (9.3%)	
Construction/Manufacturing/ Transport	6/90 (6.7%)	2/43 (4.7%)	
Education	5/90 (5.6%)	1/43 (2.3%)	
Health	24/90 (26.7%)	14/43 (32.6%)	
Hospitality/Retail	4/90 (4.4%)	1/43 (2.3%)	
Public Service	6/90 (6.7%)	4/43 (9.3%)	
Unemployed	1/90 (1.1%)	0/43 (0%)	
Other	30/90 (33.3%)	16/43 (37.2%)	
**Country of birth**			0.365
Australia	69/90 (76.7%)	37/43 (86.0%)	
United Kingdom	17/90 (18.9%)	4/43 (9.3%)	
Other	4/90 (4.4%)	2/43 (4.7%)	
**Number of admissions in previous 12 months** ^a^			0.460
One	38/90 (42.2%)	17/43 (39.5%)	
Two	21/90 (23.3%)	7/43 (16.3%)	
Three	15/90 (16.7%)	12/43 (27.9%)	
Four or more	16/90 (17.8%)	7/43 (16.3%)	
**Length of hospital admission** ^a^			0.260
Less than one week	38/90 (42.2%)	12/43 (27.9%)	
One to two weeks	27/90 (30.0%)	20/43 (46.5%)	
Two to four weeks	13/90 (14.4%)	5/43 (11.6%)	
Greater than four weeks	12/90 (13.3%)	6/43 (14.0%)	
**Prior experience reported with acute deterioration** ^a^			0.577
Yes	46/89 (51.7%)	20/43 (46.5%)	
**Prior experience reported with** ^a^ **RRT review** ^a^^,^^b^			0.430
Yes	27/89 (30.3%)	16/43 (37.2%)	

P-values determined from Fisher-Freeman-Halton Exact Test.

RRT =  Rapid Response Team.

^a^Family reporting on the patient who they were accompanying.

^b^22 of 46 respondents who had prior experience with a RRT review were identified through purposeful sampling as part of qualitative interviews completed within the wider mixed method study.

### Knowledge

#### Signs and symptoms and vital signs.

132 participants completed all questions associated with signs and symptoms and 133 completed all questions associated with vital signs. Patient and family perceptions of the signs and symptoms that they associated with acute clinical deterioration and the distribution of responses for vital signs are presented in Tables 2 and 3, respectively.

**Table 2:** Patient and family perceptions of signs and symptoms of acute deterioration (ordered by most to least frequently selected as “absolutely”).

Signs and symptoms				
Sign/symptom	Absolutely	Possibly	Definitely not	Do not know
Chest pain	115/133 (86.5%)	17/133 (12.8%)	0/133 (0%)	1/133 (0.8%)
Sudden arm, leg, or facial weakness	113/133 (85%)	17/133 (12.8%)	0/133 (0%)	3/133 (2.3%)
Sudden slurred speech	111/133 (83.5%)	21/133 (15.8%)	0/133 (0%)	1/133 (0.8%)
Unexpected or severe pain or discomfort	109/133 (82%)	22/133 (16.5%)	2/133 (1.5%)	0/133 (0%)
Signs of infection	103/133 (77.4%)	29/133 (21.8%)	0/133 (0%)	1/133 (0.8%)
Fever	86/133 (64.7%)	44/133 (33.1%)	3/133 (2.3%)	0/133 (0%)
Unexpected confusion	81/133 (60.9%)	47/133 (35.3%)	3/133 (2.3%)	2/133 (1.5%)
Chronic pain	80/132 (60.6%)	43/132 (32.6%)	8/132 (6.1%)	1/132 (0.8%)
Passing little urine (e.g., over a whole day)	78/133 (58.6%)	48/133 (36.1%)	5/133 (3.8%)	2/133 (1.5%)
Low body temperature or chills	77/132 (58.3%)	47/132 (35.6%)	2/132 (1.5%)	6/132 (4.5%)
Pale, clammy or usually cold	77/133 (57.9%)	54/133 (40.6%)	0/133 (0%)	2/133 (1.5%)
New noisy breathing	77/133 (57.9%)	50/133 (37.8%)	1/133 (0.8%)	5/133 (3.8%)
Pulse more than 120 beats a minute	68/133 (51.1%)	42/133 (31.6%)	4/133 (3.0%)	19/133 (14.3%)
Feeling faint or dizzy	65/133 (48.9%)	65/133 (48.9%)	1/133 (0.8%)	2/133 (1.5%)
Shortness of breath	64/133 (48.1%)	64/133 (48.1%)	3/133 (2.3%)	2/133 (1.5%)
Patchy or discoloured skin (e.g., red or purple patches)	61/133 (45.9%)	62/133 (46.6%)	2/133 (1.5%)	8/133 (6.0%)
Difficulty swallowing	60/133 (45.1%)	69/133 (51.9%)	0/133 (0%)	4/133 (3.0%)
Unexpected drowsiness	55/133 (41.4%)	69/133 (51.9%)	4/133 (3.0%)	5/133 (3.8%)
Swelling of the ankles	52/133 (39.1%)	72/133 (54.1%)	3/133 (2.3%)	6/133 (4.5%)
Breathing at 20 breaths a minute	32/133 (24.1%)	35/133 (26.3%)	18/133 (13.5%)	48/133 (36.1%)
Blood pressure 120/80 mmHg	15/133 (11.3%)	31/133 (23.3%)	60/133 (45.1%)	27/133 (20.3%)

NB: signs and symptoms not associated with acute clinical deterioration highlighted in grey

**Table 3:** Distribution of responses to vital signs.

Vital signs			
	SBP	HR	RR
**Mean (SD)**	120.3 (15.5)	90.3 (13.5)	23.9 (8.7)
**Median (IQR)**	117.4 (111.8-125.8)	89.6 (84.8-96.0)	20.6 (17.3-29.9)
**Range**	85-176	40-158	4-40
**Accepted range**	**>100 and < 130mmHg**	**60 to 100/minute**	**12 to 20/minute**

In examining vital signs, overall, respondents were most knowledgeable for a normal conscious state, 117/133 (88.0%) correct, and less so for heart rate 115/133 (86.5%), blood pressure 106/133 (79.7%), and respiratory rate 57/133 (42.9%). Patients, compared to family, were less knowledgeable of heart rate, with 73/90 (81.1%) correct, compared to 42/43 (97.7%), p = 0.006. Both showed similar level of knowledge for the other vital signs. Only 30% of all respondents correctly identified normal across all vital signs.

#### Overall knowledge of clinical deterioration.

A knowledge score was established for all study participants. Knowledge scores were similar for patients and family members (observed range 4-25 (from a possible score of 0 to 25); median =  14; IQR 11-17 and observed range 2-23, median =  16; IQR 11-19, respectively p = 0.28). Consumer type (patient or family), gender, educational level, number of hospital admissions, length of stay, or prior experience with either acute clinical deterioration or a RRT review did not significantly influence knowledge score. Participants aged > 80 years scored on average 4.6 points (18.4%) higher compared to respondents in the < 40 years age group (95% CI 0.55, 8.56; p = 0.026).

#### Confidence.

128 participants completed all survey items related to confidence. Observed total scores were similar between patients (observed range 23-35 (from a possible score of 7 to 35); median =  28; IQR 27-31) and family members (observed range 21-35; median =  30; IQR 27-32; p = 0.25). Responses to each question included within the scoring system are provided in S3 File. No significant differences were identified between patients and family members for any single item.

#### Factors associated with confidence.

Consumer type (patient or family), age, gender, educational level, number of hospital admissions or length of stay in the previous 12 months, and prior experience with either acute clinical deterioration or a RRT review, did not significantly influence confidence score. There was a positive association between knowledge of signs and symptoms and confidence where a 5-point (20% increase) in knowledge score was associated with a 0.94-point (3.4%) increase in confidence score (95% CI 0.28, 1.6; p = 0.006), adjusted for the factors listed above.

#### Barriers and facilitators.

133 participants completed all questions used to establish the barrier score. Scores were similar for patients (observed range 12-31 (from a possible score of 11 to 55); median =  23; IQR 20-25) and family (observed range 13-36; median =  24; IQR 22-26; p = 0.32). Responses to each item included within this score are provided in S3 File.

Of the individual items, patients, compared to family, were more likely to strongly agree with the statement “It is easier to raise concerns about my/my family member’s medical condition if the doctors and nurses ask me if I am concerned”, 28/90 (31.1%) vs 6/43 (14.0%), p = 0.026.

#### Factors associated with barrier score.

None of the factors of consumer type (patient or family), age, gender, number of hospital admissions or length of stay in the last 12 months, prior experience with either acute clinical deterioration or a RRT review significantly influenced barrier score. There was no significant correlation between knowledge of signs and symptoms and barrier scores (Pearson’s r = -0.05; p = 0.54). In contrast there was a negative association between confidence and barrier scores. For every 5-point (17.9%) increase in confidence, the barrier score decreased by 1.95-points (4.4%) (95% CI 0.8,3.1; p = 0.001).

#### Impact of barrier score on confidence and knowledge of signs and symptoms.

**Fig 3:**
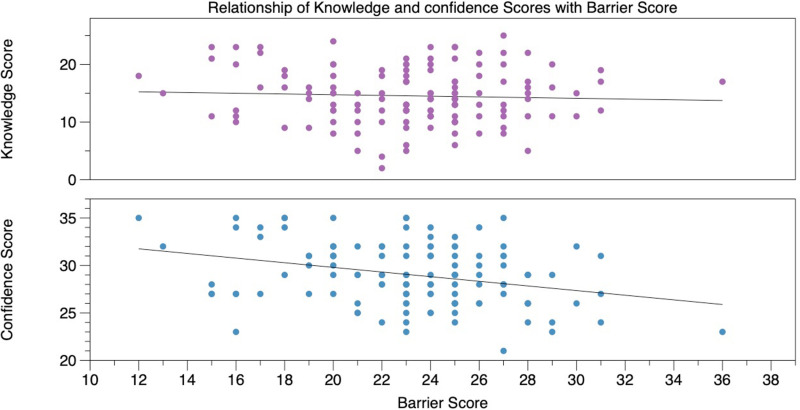
Scatterplot of observed barrier scores against knowledge scores (top) and confidence scores (bottom) with linear line of best fit.

A negative correlation (Pearson’s r = -0.31, p < 0.001) was identified between barrier and confidence scores. After controlling for potentially confounding background factors, a 5-point (11.4%) increase in barrier score was associated with a 1.3-point (4.6%) decrease in confidence (95% CI 0.6, 2.0; p = 0.001). There was no significant correlation between barrier and knowledge score (Fig 3).

#### Impact of barrier score on the relationship between knowledge and confidence.

**Fig 4:**
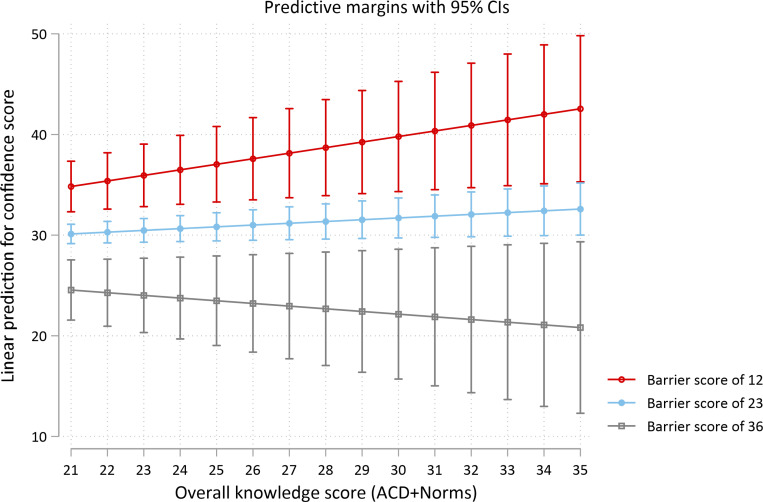
Predicted confidence scores and 95% confidence intervals for the range of observed knowledge scores for barrier scores of 12, 23 and 36. Predicted scores are from multivariable linear regression model with interaction between knowledge score and confidence score included.

The positive association between knowledge and confidence scores differed significantly depending on the barrier score (p = 0.04). The association was strongest at the lowest barrier score of 12, where a 1-point (4%) increase in knowledge was associated with a 0.6-point (2.1%) increase in confidence (95% CI 0.2, 0.9; p = 0.004), smaller at the mean barrier score of 23, where a 1-point (4%) increase in knowledge was associated with a 0.2-point (0.7%) increase in confidence (95% CI 0.1, 0.3; p = 0.006), and no longer significant, but trending in the opposite direction, at the maximum barrier score of 36, where a 1-point (4%) increase in knowledge was associated with an average 0.3-point (1.1%) decrease in confidence, p = 0.22 (Fig 4).

### Knowledge of local consumer escalation system

38/133 (28.6%) of all respondents were aware of, and 14/133 (10.5%) understood how to use the hospital’s CES, with no significant difference between patients and family. The primary source of CES information for respondents was brochures, posters and digital displays within the hospital (29/133; 21.8%), whilst only 4/133 (3.0%) had been informed by staff. Based on whether a respondent was aware of the local CES there was no significant difference in their overall knowledge score (aware of CES median knowledge score =  15, unaware of CES median knowledge score =  14; p = 0.46), their confidence score (aware of CES median confidence score =  29, unaware of CES median confidence score =  29; p = 0.68), or their barrier score (aware of CES median barrier score =  23, unaware of CES median barrier score =  24; p = 0.89).

During the study period, there were no documented cases of consumer escalation reaching the RRT using the hospital’s CES.

## Discussion

Our findings suggested that limitations existed in overall consumer knowledge of acute clinical deterioration signs and symptoms and that awareness of the local CES was low. Collectively, these knowledge gaps may limit patient and family interpretation of symptom relevance, their response, and their ability to escalate concerns. An association was noted between knowledge and confidence; however, the effect diminished, and was negated, as the level of perceived barriers grew.

If CES are to reach their full safety potential, efforts to address knowledge, confidence, and barriers are essential. Multifaceted interventions including dedicated educational strategies, facilitation of staff-patient/family engagement, and organisational support are required. Addressing health literacy associated with CES (at both individual and health service levels) is fundamental to all aspects.

### Knowledge of clinical deterioration

Prior research has explored consumer’s self-reported perceptions of either their own medical knowledge [29], ability to recognise their clinical change [11,29–33], sign and/or symptoms at the time of deterioration [29,30], what they would look for to identify a patient was becoming more unwell [33], and efficacy to report symptoms [11,29–31,33]. We examined the signs and symptoms that patients and family members associated with, and thus their knowledge of, acute clinical deterioration. Our findings suggested that healthcare consumers did not share a universal understanding. Knowledge limitations were noted, particularly around sepsis. Furthermore, knowledge of vital signs varied widely. Given the difficulties that clinicians face in recognising deterioration, including with vital signs such as the respiratory rate [34], the knowledge gap identified is not a criticism of healthcare consumers. Nor does it represent an expectation that patients and family knowledge be measured against that of staff. It also does not underestimate the unique knowledge that patients and families may have of themselves or loved ones that may be of value in identifying subtle changes. Finally, we cannot deny that for some individuals, stating that a sign or symptom is possibly an indication of acute clinical deterioration may be an appropriate response for them given contextual factors. Nonetheless, our findings are in keeping with the wider literature that have identified limitations in public awareness of signs and symptoms associated with out-of-hospital time critical conditions [35,36]. Such studies have demonstrated how knowledge gaps can impact on public understanding about severity of illness and the importance of seeking urgent assistance resulting in delays in obtaining medical input [35,36]. This impact of knowledge is arguably also relevant within the inpatient setting.

Further to the above, previous research associated with CES has demonstrated that patients rely upon symptoms [30], physical or behavioural signs [33], or an awareness of alteration from their normal [29] to identify clinical change. However, qualitative studies post deterioration have noted that the utility of this awareness has been limited by difficulties in interpreting significance [29,30] and attributable cause [29]. Our findings suggested that knowledge limitations have the potential to impact on consumer recognition of, and response to, clinical deterioration, including their decision of whether they should inform staff and potentially to continue to escalate if they feel unheard. Ultimately, these knowledge limitations may impact on the performance of, and reliance upon, CES. The importance of increasing public awareness of signs and symptoms associated with time dependent medical condition has become a key strategy within public health campaigns [21,22]. A similar argument could exist for inpatient acute clinical deterioration. Our results identify areas to focus upon within future education about clinical deterioration that is necessary if CES are to be both effective in enhancing patient safety and efficient in resource utilisation.

Prior research has suggested that patients with chronic conditions may be more attentive in self-monitoring and identifying alterations [29]. It has also been noted that prior experiences and hospitalisation may assist patients in identifying symptoms [30], parents to notice clinical change [37], and consumers to more readily escalate concerns [37,38]. We; however, did not identify a significant impact of prior hospitalisation or experience with clinical deterioration on study measures. Given that confidence can take time to develop [37], it may require longer than the 12-month period utilised within our study to see a significant effect from prior experience on the factors we investigated.

### Knowledge of consumer escalation system

Within our study period, both awareness and understanding of the CES was poor. This limited awareness and understanding is a barrier to CES use and effectiveness. Within the literature, reported, awareness of CES vary substantially[9]but are highest amongst services that implemented dedicated awareness-raising strategies [3,39,40]. The health service in which this study was conducted has since taken steps to raise greater patient, family, and staff awareness.

The way in which patients and families are informed about CES has the potential to impact on understanding, awareness, and system use [3,40,41]. The leading source of CES information reported by respondents within our study was displays within the hospital and few were informed directly by staff. Prior research has indicated that a multimodal, active, and ongoing approach is required [3,11,41,42] with information provided by staff being a crucial component [40–42]. Given the high demand on clinicians, further investigation is needed to determine the most efficient and sustainable way of achieving this information provision.

The low levels of patient and family awareness about CES also highlight the importance of considering the health literacy environment (not just individual health literacy) [43]. The health literacy environment includes aspects about how the health system can impact on how patients and family members access, evaluate, and utilise health associated information and services [43]. Accessible and understandable information is crucial [43] to allow patients and families to understand the CES purpose, make informed decisions about system use, and to escalate concerns.

### Confidence

In establishing an overall confidence score, we explored patient and family self-perceived knowledge and ability to recognise clinical change, communication with staff, and an escalation hierarchy.

With self-perceived knowledge, most respondents indicated confidence in noticing a change in their own or loved one’s condition and that they had sufficient understanding to inform staff. Whilst our findings align with that of King et al. [33], they contrast with other research suggesting patients perceive limitations in their knowledge to use CES [29]. Our results may reflect the nature of our study sample.

Our survey participants were English speaking. It is essential that future research considers those with limitations in language proficiency and from non-English speaking and culturally and linguistically diverse backgrounds. Such individuals are more likely to have lower levels of health literacy, lower sense of empowerment, and be at greater risk of experiencing adverse events in healthcare [44]. Differences have also been noted in the information that individuals within these groups are typically provided and their communication, interactions, and engagement with staff [44]. Finally, they may hold differing beliefs about illness, treatment, and the role of family [44]. All of these factors are of significant relevance in the context of CES.

Amongst our study participants, willingness to communicate concerns about clinical deterioration decreased as the level of escalation increased and was influenced by how receptive respondents perceived staff to be. Our outcomes are in keeping with other studies reporting a reluctance from healthcare consumers to escalate beyond a patient’s immediate treating team [29,30] and reduced confidence to bypass staff and use CES [11,39]. Such reluctance and reduced confidence requires consideration in the design and implementation of CES. Our findings highlight the important role that staff have in supporting patient and family engagement in safety practices and healthcare. Facilitating information provision from staff about CES (as has been noted above) and routine enquiry between staff and healthcare consumers about clinical concerns, as is discussed further in relation to barriers, are potential strategies. Educational efforts may also have an important role [31,33,45]. Prior research has suggested increased healthcare consumer awareness of the need to report concerns of deterioration [33] and self-efficiency to do so [31] after the introduction of educational materials [33] and intervention [31]. However, based on the limitations reported in prior studies [31,33], further research continues to be warranted.

### Barriers and facilitators

A complex interplay of factors impacts upon clinicians identifying and responding to deterioration [34]. It is therefore reasonable to consider that patients and families will similarly face barriers.

The attitudes and actions of hospital staff are key areas that can influence CES performance [14,46]. We found study respondents’ perception of staff willingness to initiate discussions about patient and family concerns, and to listen to and value their concerns were significant enablers. Our findings therefore agree with prior studies [29,30] that constructive staff actions and a positive patient/family-staff relationship have the potential to reduce barriers for consumer escalation. Suggestion exists for the value of staff routinely asking patients and family about perceived levels of wellness [47] or clinical concerns [30]. Fostering such routine engagement may help to facilitate open communication [47], increase the potential that patients and family will express apprehensions [30], and support the early identification of clinical deterioration [30,47]. These interactions may also help to identify other issues not related to clinical deterioration that can be addressed through channels other than resource intensive CES [47]. In examining frequency of staff enquiring with patients and family, the first component of the newly introduced Martha’s Rule in the United Kingdom includes staff asking patients about how they are feeling and any noticed clinical change at least daily [7]. Other research studies have noted enquiring at the time of completing routine clinical observations [47]. Our study supports the need to introduce such regular enquiry; however, we cannot shed light on the optimal and most sustainable frequency, which is still to be determined. Education to raise staff awareness of the value in their interactions with patients and family, shifting in hospital culture to support this practice, and health service organisational support will be essential to achieving this change.

Overall, our study participants held strong beliefs about their right to inform staff of their concerns about deterioration. Although many studies have documented consumer apprehension to questioning clinicians [32,38,39,48], or concerns of damaging their relationship with staff [11,48], being negatively judged or labelled [37,48], treated differently [48], and imposing upon already strained services [29,32,40], our findings suggested that these were least frequent of the barriers. That noted, there was still almost one in 10 respondents who indicated apprehension that escalating concerns would have a negative impact on their own or family member’s care. Given the nature of our sample, this figure may be higher, particularly within vulnerable groups. Again, staff routinely enquiring with patients and family about clinical concerns and fostering a health service culture that promotes patient and family engagement may be initial steps in addressing such apprehension.

Other more prominent barriers within our study included a reliance by patients and families on the clinician’s ability to detect deterioration and the perception of clinicians knowing “what is best” for them. Whilst this reliance and perception may reflect the degree of trust that consumers have in healthcare professionals [30], it may also indicate a preference towards a passive role during hospitalisation [30]. Targeting knowledge gaps and enhancing communication and consumer-staff relationship may increase consumer participation in care [49] and in turn, improve CES performance.

### Implications

If CES are to achieve their intended purpose, empowering patients and families to use consumer escalation processes appropriately and effectively is essential. Our findings of a positive association between increased knowledge and confidence therefore have important implications about where health services can target their efforts. Our findings support the work of others looking at educational interventions to increase patient [31,33,45] and family [33,45] confidence to recognise and inform staff about concerns of clinical deterioration. Our results support the importance of extending education to a patient’s family members or carers and that a similar approach could be taken to engage and educate this group. Ultimately our findings around knowledge and confidence relate back to health literacy associated with CES and, as has been recognised by others [33], educational efforts will need wider support through hospital policy and staff actions.

It is important to note that we also identified that the positive association between knowledge and confidence can be eroded, and potentially negated, as barriers increase. Thus, endeavours to increase knowledge should be accompanied by efforts to reduce barriers to consumer escalation. With organisational support, staff, including through their interactions with patients and family, are in a key role to help address barriers.

### Limitations and strengths

A number of factors must be acknowledged that may limit the generalisability of our study results and confidence in findings.

Outside of the CROSS reporting checklist, the study was not based upon another pre-existing framework.

Whilst our survey tool was developed through a multistage process with consumer input, and was pilot tested with consumers prior to use, the tool did not undergo further formal reliability assessment, psychometric analysis, or validation processes.

In examining knowledge, our measure focused on signs and symptoms. We did not examine the unique insight of patients and families in relation to their knowledge of their own or loved one’s normal self that may be of importance in the identification of subtle changes. In looking at barriers, our questions focused on individual determinants and perceptions about the impact of staff response and actions. Further research is required to examine the impact of situational factors (such as those associated with context and access to resources) and healthcare service and organisational considerations, including ward and hospital culture.

Our sample was drawn from a single healthcare facility. Whilst our sample size was determined by available resources, an undersized sample cannot provide conclusive results [26]. At the time at which this research was conducted, studies that exceeded our sample adopted less extensive survey tools or focused on the analysis of CES data. Most other work had been in-depth qualitative studies or centred upon evaluating system implementation within individual facilities. There had only been one small scale randomised control trial investigating the impact of a patient education intervention directed at enhancing self-efficacy to identify and report symptoms [31]. More recently, since the completion of our study, a larger scale paper has emerged examining the effectiveness of educational efforts to increase patient and family knowledge and confidence [33]. Nonetheless, further studies with sample sizes based on power analysis calculations are required.

Regarding our sampling method, nonprobability sampling creates a potential source of bias and limits the generalisability of results and confidence in the statistical inferences made [26]. The impact of COVID-19 visitor restrictions may have influenced our sample distribution where patients outnumbered family.

Our study population was limited to English speaking adults. We recognise that non-English speakers are important users of the health service. Overall, approximately 44 per cent of our respondents reported having a university degree, exceeding the national average reported in Australian Bureau of Statistics data of 33 per cent for 15 to 74 year olds [50]. Focus was also upon the adult populations and therefore our findings may not extend to the paediatric setting or the views of children or adolescent family members, including those who are carers for adults. Finally, all participating patients were medically stable and physically and cognitively well enough to voice opinions. In practice, however, there will inherently be patients who, owing to the severity of their condition, will be dependent on others to identify deterioration and escalate on their behalf [30].

Finally, awareness levels of our local CES were low and results may differ in sites where awareness is greater, or a different CES model exists.

Regarding strengths, we collected information from both patients and families using a similar survey, thus allowing comparisons to be made. Surveys were completed across multiple hospital wards, ensuring that a broad case-mix was captured within a facility that has an established CES. Finally, we explored, and reported upon, the potential impact of a wide array of demographic and prior experience related aspects.

### Recommendations for future research

Our study provides direction for developing interventions focused on increasing patient and family health literacy in association with clinical deterioration and escalating concerns. Specifically, the need to increase awareness of clinical deterioration and consumer escalation processes. We have also highlighted potential barriers to address and the crucial role that staff have in supporting CES. Future research is required to design and test the effectiveness of such interventions. It is also imperative that future studies include those from vulnerable populations, including culturally and linguistically diverse backgrounds, with sufficiently powered sample sizes.

## Conclusion

CES are an important initiative that have been introduced to increase patient safety. However, we identified limitations in patient and family knowledge that may impact on the ability of CES to achieve their goal. Consideration needs to be given to patient and family health literacy in association with acute clinical deterioration and escalation processes and that of the healthcare service. Thought also needs to be directed to how the presence of barriers to identifying deterioration and voicing concerns may impact on both consumer confidence and CES success. Our findings provide guidance for the development of future interventions to help address such knowledge gaps and barriers. However, such interventions will need to recognise the potential impact of factors at consumer, staff, and system levels if patient safety outcomes are to be truly promoted.

## Supporting information

S1 File Surveys. (DOCX)

S2 File Scoring systems. (DOCX)

S3 File Participant responses to confidence and barrier associated questions. (DOCX)

S4 File Unadjusted statistical analysis. (DOCX)
