# Supplementary material for: A cross-sectional survey analysis of patient and family knowledge, confidence, and perceived barriers to reporting patient deterioration
Source: PLoS One. 2025 Mar 11;20(3):e0319546. doi: 10.1371/journal.pone.0319546 (PMC11896061; doi:10.1371/journal.pone.0319546)
Supplement: S1 File — (DOCX) [file pone.0319546.s001.docx]

**Supporting File S1: Surveys**

**Patient survey**

Sometimes, the medical condition of a patient in hospital suddenly or unexpectedly worsens. This is called acute clinical deterioration. We would like to explore your knowledge about this topic and your confidence to tell staff if you felt that your medical condition had suddenly or unexpectedly worsened during your hospital stay. We are interested in your opinion rather than right or wrong answers.

1. From the list below, please indicate those that you consider to be a sign or symptom of

clinical deterioration.

| Sign/symptom | Definitely not | Possibly | Absolutely | Don’t know |
| --- | --- | --- | --- | --- |
| Shortness of breath |  |  |  |  |
| Pulse more than 120 beats a  minute |  |  |  |  |
| Unexpected drowsiness |  |  |  |  |
| Unexpected confusion |  |  |  |  |
| Fever |  |  |  |  |
| Low body temperature or chills |  |  |  |  |
| Breathing at 20 breaths per minute |  |  |  |  |
| Feeling faint or dizzy |  |  |  |  |
| Unexpected or severe pain |  |  |  |  |
| Chronic pain |  |  |  |  |
| Passing little urine (e.g., over a  whole day) |  |  |  |  |
| Patchy or discoloured skin (e.g.,  new red or purple patches) |  |  |  |  |
| Pale, clammy, or unusually cold  Skin |  |  |  |  |
| New noisy breathing |  |  |  |  |
| Sudden arm, leg or facial  Weakness |  |  |  |  |
| Chest pain |  |  |  |  |
| Blood pressure 120/80 mmHg |  |  |  |  |
| Difficulty swallowing |  |  |  |  |
| Breathing at 20 breaths a  minute |  |  |  |  |
| Sudden slurred speech |  |  |  |  |
| Ankle swelling |  |  |  |  |
| Signs of infection |  |  |  |  |

2. During my hospital stay, if I had concerns that my medical condition had suddenly or unexpectedly worsened, I would (select one only):

€ Say nothing

€ Only tell a family member or close friend (and not the hospital staff)

€ Wait to see if I improved on my own before telling a doctor or nurse

€ Wait until a doctor or nurse came to see me before raising my concerns

€ Tell a doctor or nurse immediately (e.g., press the call bell and tell them)

3. Please indicate how strongly you agree or disagree with the following statements when in

hospital, as a patient.

|  | Strongly disagree | Disagree | Neutral | Agree | Strongly agree |
| --- | --- | --- | --- | --- | --- |
| In regard to my health, I know what is 'normal' for me |  |  |  |  |  |
| I am confident that I would notice if my medical condition suddenly or unexpectedly worsened |  |  |  |  |  |
| I rely on the doctors and nurses to notice if my medical condition is deteriorating |  |  |  |  |  |
| I have always felt safe as a patient when in hospital |  |  |  |  |  |

4. Imagining that you had a concern about a sudden or unexpected change in your medical condition during your hospital stay, please indicate how you feel about the following statements.

|  | Strongly disagree | Disagree | Neutral | Agree | Strongly agree |
| --- | --- | --- | --- | --- | --- |
| As a patient, I have the right to  tell staff if I have concerns about  any sudden or unexpected  deterioration in my medical  condition |  |  |  |  |  |
| I would feel confident to tell a  doctor or nurse of my concerns  about a change in my medical  condition |  |  |  |  |  |
| If the concerns I voiced were not  addressed, I would ask to speak  to a more senior nurse or doctor |  |  |  |  |  |
| If my concerns about a sudden  or unexpected change in my  medical condition were still not  addressed, I would ask for the  hospital's Medical Emergency  Response (MER) team to be  called |  |  |  |  |  |
| I feel that there is no need for  me to raise concerns because  the staff would be able to notice  if there was a change in my  condition |  |  |  |  |  |
| I feel there is no need for me to  raise concerns because the staff  would know what is best for me |  |  |  |  |  |

5. When I am in hospital, I feel confident to raise concerns that I may have about a sudden or unexpected deterioration in my medical condition with the doctors and nurses, because: (you can tick more than one):

€ The experience I have with a chronic medical condition

€ The knowledge I have gained from previous hospital admissions

€ My personality or natural tendencies

€ My education or work background

€ The good relationship I have with my doctors and nurses

€ My belief that medical and nursing staff would listen to and act upon my concerns

€ None of the above - I do not feel confident

6. Please indicate how you feel about the following statements

|  | Strongly disagree | Disagree | Neutral | Agree | Strongly agree |
| --- | --- | --- | --- | --- | --- |
| It is easier to raise concerns  about my medical condition, if  the doctors and nurses ask me if  I am concerned |  |  |  |  |  |
| It is easier to raise my concerns  when I feel that the doctors and  nurses value my opinion |  |  |  |  |  |
| I do not feel confident to raise  my concerns with doctors and  nurses who do not usually look  after me |  |  |  |  |  |
| I find it difficult to communicate  changes in my medical condition  to the doctors and nurses |  |  |  |  |  |
| I feel that I do not have sufficient  medical knowledge to let the  doctors and nurses know of any  sudden or unexpected  deterioration in my medical  condition |  |  |  |  |  |
| I am worried that if I expressed  concerns about a sudden or  unexpected change in my  medical condition I will upset or  unnecessarily disturb the  doctors and nurses |  |  |  |  |  |
| I am worried that if I express  concerns about my medical  condition, I will be negatively  judged |  |  |  |  |  |
| I am worried that raising my  concerns may have a negative  impact upon my care |  |  |  |  |  |
| I am worried that if I express  concerns about my medical  condition, I will get the junior  nurses and doctors into trouble |  |  |  |  |  |

7. When in hospital as a patient (either this admission or a previous hospital stay) have you ever experienced a sudden or unexpected worsening in your medical condition?

€ Yes

€ No

If yes, was it because of problems with (can tick more than one response):

€ Breathing

€ Pulse/heart rate

€ Blood pressure

€ High or Low temperature

€ Drowsiness or consciousness

€ Confusion

€ Infection

Were there any delays in staff responding to you when you became acutely unwell?

€ Yes

€ No

How would you rate the overall quality of care that you received during this time?

€ Unacceptable

€ Poor

€ Average

€ Good

€ Excellent

8. When in hospital as a patient the doctors and nurses record a number of observations (your vital signs) from you. Please indicate what you consider to be a normal (It is OK to guess if you are unsure):

- Systolic (upper value) blood pressure (scale range of 60 to 200mmHg)
- Pulse per minute (scale range of 40 to 200 per minute)
- Number of breathes per minute (scale range of 4 to 40 per minute)
- For someone who appears to be asleep (options of a. Unable to wake at all, b. Wakes only when pain applied (e.g., squeeze of shoulder), c. Wakes briefly (for less than 10 secs) when spoken to but then falls asleep, d. Wakes easily when spoken to and stays awake

9. Are you aware that hospitals have Medical Emergency Response Teams (or Rapid Response Teams)? These are a team of emergency/critical care trained doctors and nurses, who respond immediately to patients experiencing a sudden or unexpected deterioration in their medical condition.

€ Yes

€ No

10. Have you ever been attended by a Medical Emergency Response Teams (or Rapid Response Teams)?

€ Yes

€ No

If yes, how did you feel about this experience (can select more than one option)?

€ I felt frighten

€ I felt worried

€ I felt unsafe

€ I felt safe

€ I felt reassured

€ I don't remember the experience

11. Are you aware that this hospital offers a process called 'You're Worried, We're Listening'?

€ Yes

€ No

Do you know how to use this process if you have a concern?

€ Yes

€ No

If yes, how did you learn about this process? (You can select more than one option)

€ Information provided to me before my admission to hospital

€ My doctor or nurse told me about this process during this admission

€ I saw information about this process within the hospital

€ I heard about it from others and/or the media

12. What gender do you identify with?

€ Female

€ Male

€ Other

13. What is your age group?

€ < 30

€ 31-40

€ 41-50

€ 51-60

€ 61-70

€ 71-80

€ >80

14. What is the highest level of education that you have completed?

€ Primary

€ Secondary

€ Vocational/Apprenticeship

€ University

15. What area do you work in, or the main area that you have previously worked in?

€ Agriculture Education

€ Health

€ Home duties

€ Hospitality/Retail

€ Information Technology

€ Public service

€ Sport/recreation

€ Business/Trade/Office

€ Construction/manufacturing/transport

€ Retired

€ Unemployed

€ Other

16. In what country were you born?

€ Australia

€ China

€ Germany

€ Greece

€ India

€ Italy

€ Malaysia

€ New Zealand

€ Philippines

€ South Africa

€ United Kingdom

€ Vietnam

€ Other

17. In past 12 months, how many times have you been admitted to hospital?

€ None

€ Once

€ Twice

€ Three times

€ Four times

€ Five times

€ Six or more

18. Approximately how long was your longest stay in hospital?

€ Less than one week

€ One to two weeks

€ Two to four weeks

€ More than four weeks

19. If you have any other comments or information that you would like us to know, please note them below.

**Family/carer survey**

Sometimes, the medical condition of a patient in hospital suddenly or unexpectedly worsens. This is called acute clinical deterioration. We would like to explore your knowledge about this topic and your confidence to tell staff if you became concerned that the medical condition of your family member suddenly or unexpectedly worsened during your hospital stay. We are interested in your opinion rather than right or wrong answers.

A ”family member” can be a relative, or a non-related carer, who is well known to the patient.

1. From the list below, please indicate those that you consider to be a sign or symptom of acute clinical deterioration whilst in hospital.

| Sign/symptom | Definitely not | Possibly | Absolutely | Don’t know |
| --- | --- | --- | --- | --- |
| Shortness of breath |  |  |  |  |
| Pulse more than 120 beats a  Minute |  |  |  |  |
| Unexpected drowsiness |  |  |  |  |
| Unexpected confusion |  |  |  |  |
| Fever |  |  |  |  |
| Low body temperature or chills |  |  |  |  |
| Breathing at 20 breaths per minute |  |  |  |  |
| Feeling faint or dizzy |  |  |  |  |
| Unexpected or severe pain |  |  |  |  |
| Chronic pain |  |  |  |  |
| Passing little urine (e.g., over a  whole day) |  |  |  |  |
| Patchy or discoloured skin (e.g.,  new red or purple patches) |  |  |  |  |
| Pale, clammy, or unusually cold  Skin |  |  |  |  |
| New noisy breathing |  |  |  |  |
| Sudden arm, leg or facial  Weakness |  |  |  |  |
| Chest pain |  |  |  |  |
| Blood pressure 120/80 mmHg |  |  |  |  |
| Difficulty swallowing |  |  |  |  |
| Breathing at 20 breaths a  Minute |  |  |  |  |
| Sudden slurred speech |  |  |  |  |
| Ankle swelling |  |  |  |  |
| Signs of infection |  |  |  |  |

2. During my family member’s hospital stay, if I had concerns that their medical condition had suddenly or unexpectedly worsened, I would (select one only):

€ Only tell another family member/friend (and not hospital staff)

€ Wait to see if they improved on their own before telling a doctor or nurse

€ Wait until a doctor or nurse came to see my family member before raising my concerns

€ Tell a doctor or nurse immediately

€ Say nothing

3. Please indicate how strongly you agree or disagree with the following statements when your family member is in hospital as a patient.

|  | Strongly disagree | Disagree | Neutral | Agree | Strongly agree |
| --- | --- | --- | --- | --- | --- |
| In regard to my family member’s health, I know what is 'normal' for them |  |  |  |  |  |
| I am confident that I would notice if my family member’s medical condition suddenly or unexpectedly worsened |  |  |  |  |  |
| I rely on the doctors and nurses to notice if my family member’s medical condition is deteriorating |  |  |  |  |  |
| I have always felt that my family member is safe when in hospital as a patient |  |  |  |  |  |

4. Imagining that you had a concern about a sudden or unexpected change in your family member’s health during their hospital stay, please indicate how you feel about the following statements.

|  | Strongly disagree | Disagree | Neutral | Agree | Strongly agree |
| --- | --- | --- | --- | --- | --- |
| As a patient’s family member, I have the right to tell staff if I have concerns about any sudden or unexpected deterioration in my family member’s medical condition |  |  |  |  |  |
| I would feel confident to tell a  doctor or nurse of my concerns  about a change in my family member’s medical condition |  |  |  |  |  |
| If the concerns I voiced were not  addressed, I would ask to speak  to a more senior nurse or doctor |  |  |  |  |  |
| If the concerns I voiced were still not addressed, I would ask for the  hospital's Medical Emergency  Response team to be called |  |  |  |  |  |
| I feel that there is no need for  me to raise concerns because  the doctors and/or nurses would be able to notice if there was a change in my family member’s medical condition |  |  |  |  |  |
| I feel there is no need to raise my concerns because the doctors and nurses know what is best for my family member |  |  |  |  |  |

5. Please indicate how much you agree with the following statements, with respect to your family member’s medical condition

|  | Strongly disagree | Disagree | Neutral | Agree | Strongly agree |
| --- | --- | --- | --- | --- | --- |
| It is easier to raise concerns if the staff ask me if I have any concerns |  |  |  |  |  |
| It is easier to raise my concerns  when I feel that the doctors and  nurses value my opinion |  |  |  |  |  |
| I do not feel confident to raise  my concerns with staff who do not usually look after my family member |  |  |  |  |  |
| I find it difficult to communicate  changes in my family member’s medical condition to the doctors and nurses |  |  |  |  |  |
| I feel that I do not have sufficient  medical knowledge to let the  doctors and nurses know of any  sudden or unexpected  deterioration in my family member’s medical condition |  |  |  |  |  |
| I am worried that I will upset the doctors and nurses if I express concerns about any sudden or  unexpected change in my family member’s medical condition |  |  |  |  |  |
| I am worried that if I express  concerns about my family member’s medical  condition, I will be negatively  judged |  |  |  |  |  |
| I am worried that raising my  concerns may have a negative  impact upon my family member’s care |  |  |  |  |  |
| I am worried that if I express  concerns about my family member’s medical condition, I will get the junior nurses and doctors into trouble |  |  |  |  |  |

6. When in hospital as a patient (either this admission or a previous hospital stay) has your family member ever experienced a sudden or unexpected worsening in their health?

€ Yes

€ No

If yes, was it because of problems with (can choose more than one):

€ Breathing

€ Pulse/heart rate

€ Blood pressure

€ High or Low temperature

€ Drowsiness or consciousness

€ Confusion

€ Infection

€ Unknown

Were there any delays in a doctor or nurse responding to your family member when they became acutely unwell?

€ Yes

€ No

How would you rate the overall quality of care that your family member received during this time?

€ Unacceptable

€ Poor

€ Average

€ Good

€ Excellent

7. When your family member is a patient in hospital, the doctors and nurses record a number of observations (your vital signs) from them. Please indicate what you consider to be a normal (It is OK to guess if you are unsure):

- Systolic (upper value) blood pressure (scale range of 60 to 200mmHg)
- Pulse rate (scale range of 40 to 200 per minute)
- Number of breathes per minute (scale range of 4 to 40 per minute)
- For someone who appears to be asleep (options of a. Unable to wake at all, b. Wakes only when pain applied (e.g., squeeze of shoulder), c. Wakes briefly (for less than 10 secs) when spoken to but then falls asleep, d. Wakes easily when spoken to and stays awake

8. Are you aware that hospitals have Medical Emergency Response Teams (or Rapid Response Teams), which are a team of critical care trained doctors and nurses who can respond immediately to any severe acute clinical deterioration?

€ Yes

€ No

9. Has your family member ever been attended by a Medical Emergency Response Team (or Rapid Response Team)?

€ Yes

€ No

If yes, how did you feel about this experience (can select more than one)?

€ I felt frighten

€ I felt worried

€ Felt unsafe

€ Felt safe

€ Felt reassured

€ Don’t recall

10. Are you aware that this hospital offers a process called 'You're Worried, We're Listening'?

€ Yes

€ No

Do you know how to use this process if you have a concern?

€ Yes

€ No

If yes, how did you learn about this process? (can select more than one option)

€ Education prior to admission

€ My family member’s doctor or nurse told me about this process during this admission

€ I saw information about this process within the hospital

€ I heard about it from others and/or the media

11. What gender do you identify with?

€ Female

€ Male

€ Other

12. Age?

€ < 30

€ 31-40

€ 41-50

€ 51-60

€ 61-70

€ 71-80

€ >80

13. What is the highest level of education that you have completed?

€ Primary school

€ Secondary school

€ Vocational/apprenticeship

€ University

14. What area do you work in?

€ Agriculture

€ Business/Trade/Office

€ Construction/manufacturing/transport

€ Education

€ Health

€ Home duties

€ Hospitality/Retail

€ Information Technology

€ Public service

€ Retired

€ Sport/recreation

€ Unemployed

€ Other

15. In what country were you born?

€ Australia

€ China

€ Germany

€ Greece

€ India

€ Italy

€ Malaysia

€ New Zealand

€ Philippines

€ South Africa

€ United Kingdom

€ Vietnam

€ Other

16. In past 12 months, how many times has a family member been admitted to hospital?

€ None

€ Once

€ Twice

€ Three times

€ Four times

€ Five times

17. Approximately how long did they stay in hospital in their last admission?

€ Less than one week

€ One to two weeks

€ Two to four weeks

€ More than four weeks

18. If you have any other comments or information that you would like us to know, please note them below.
