# Supplementary material for: A cross-sectional survey analysis of patient and family knowledge, confidence, and perceived barriers to reporting patient deterioration
Source: PLoS One. 2025 Mar 11;20(3):e0319546. doi: 10.1371/journal.pone.0319546 (PMC11896061; doi:10.1371/journal.pone.0319546)
Supplement: S2 File — (DOCX) [file pone.0319546.s002.docx]

**Supporting File S2: Scoring systems**

| **Knowledge scoring system** | |
| --- | --- |
| **Sign/symptom associated with acute clinical deterioration** | **Correct responses = 1 point for each item** |
| Shortness of breath | “Absolutely” |
| Pulse more than 120 beats a minute | “Absolutely” |
| Unexpected drowsiness | “Absolutely” |
| Unexpected confusion | “Absolutely” |
| Fever | “Absolutely” |
| Low body temperature or chills | “Absolutely” |
| Feeling faint or dizzy | “Absolutely” |
| Unexpected or severe pain or discomfort | “Absolutely” |
| Passing little urine (e.g., over a whole day) | “Absolutely” |
| Patchy or discoloured skin (e.g., red or purple patches) | “Absolutely” |
| Pale, clammy or usually cold | “Absolutely” |
| New noisy breathing | “Absolutely” |
| Sudden arm, leg, or facial weakness | “Absolutely” |
| Sudden slurred speech | “Absolutely” |
| Chest pain | “Absolutely” |
| Signs of infection | “Absolutely” |
| Difficulty swallowing | “Absolutely” |
| Swelling of the ankles | “Absolutely” |
| Chronic pain | “Definitely not” |
| Blood pressure 120/80 mmHg | “Definitely not” |
| Breathing at 20 breaths a minute | “Definitely not” |
| **Vital signs** | **Response within accepted range = 1 point** |
| Systolic blood pressure | ≥100 mmHg &≤130 mmHg |
| Pulse rate | 60 to 100 bpm |
| Respiratory rate | 12 – 20 breaths/min |
| Consciousness | “Wakes easily when spoken to and stays awake” |
| Potential survey responses to signs and symptoms of deterioration included “Absolutely”, “Possibly”, “Definitely not”, and “Don’t know”.  For participants who provided responses to all associated questions, individual survey participant responses were summed together to provide a knowledge score.  Potential scoring range = 0 to 25 (where 0 reflected the lowest level of knowledge and 25 reflected the highest level of knowledge). | |

| **Confidence scoring system** | |
| --- | --- |
| **Confidence item** | **Scoring associated with Likert response** |
| I am confident that I would notice if my/my family member’s medical condition suddenly or unexpectedly worsened  I would feel confident to tell a doctor or nurse of my concerns about a change in my/my family member's medical condition  If the concerns I voiced were not addressed, I would ask to speak to a more senior nurse or doctor  If my concerns about a sudden or unexpected change in my medical condition were still not addressed, I would ask for the hospital's Medical Emergency Response (MER) team to be called | Strongly disagree = 1    Disagree = 2  Neutral = 3  Agree = 4  Strongly agree = 5 |
| I do not feel confident to raise my concerns with doctors and nurses who do not usually look after me/my family member.  I find it difficult to communicate changes in my/family member's medical condition to the doctors and nurses.  I feel that I do not have sufficient medical knowledge to let the doctors and nurses know of any sudden or unexpected deterioration in my/family member's medical condition | Strongly disagree = 5  Disagree = 4  Neutral = 3  Agree = 2  Strongly agree = 1 |
| The scoring system was applied to each individual confidence question.  For participants who provided responses to all associated questions, individual survey participant responses were then summed together to provide a confidence score.  Potential scoring range = 7 to 35 (where 7 reflected the lowest level of confidence and 35 reflected the highest level of confidence). | |

| **Barrier scoring system** | |
| --- | --- |
| **Barrier item** | **Scoring associated with Likert response** |
| It is easier to raise concerns about my medical condition, if the doctors and nurses ask me if I am concerned    It is easier to raise my concerns when I feel that the doctors and nurses value my opinion    In regard to my/family member's health, I know what is 'normal' for me/them    As a patient/family member, I have the right to tell staff if I have concerns about any sudden or unexpected deterioration in my/family member's medical condition | Strongly disagree = 5    Disagree = 4    Neutral = 3    Agree = 2    Strongly agree = 1 |
| I am worried that if I expressed concerns about a sudden or unexpected change in my/family member's medical condition I will upset or unnecessarily disturb the doctors and nurses    I am worried that if I express concerns about my/my family member’s medical condition, I will be negatively judged    I am worried that raising my concerns may have a negative impact upon my/family member's care    I am worried that if I express concerns about my/family member's medical condition, I will get the junior nurses and doctors into trouble    I feel that there is no need for me to raise concerns because the staff would be able to notice if there was a change in my/family member's condition    I feel there is no need for me to raise concerns because the staff would know what is best for me/my family member    I rely on the doctors and nurses to notice if my/family member's medical condition is deteriorating | Strongly disagree = 1    Disagree = 2    Neutral = 3    Agree = 4    Strongly agree = 5 |
| The scoring system was applied to each individual barrier question.  For participants who provided responses to all associated questions, individual survey participant responses were then summed together to provide perceived barrier score.  Potential scoring range = 11 to 55 (where 11 reflected the lowest level of perceived barrier and 55 reflected the highest level of perceived barrier) | |
