# Supplementary material for: A cross-sectional survey analysis of patient and family knowledge, confidence, and perceived barriers to reporting patient deterioration
Source: PLoS One. 2025 Mar 11;20(3):e0319546. doi: 10.1371/journal.pone.0319546 (PMC11896061; doi:10.1371/journal.pone.0319546)
Supplement: S3 File — (DOCX) [file pone.0319546.s003.docx]

**Supporting File S3: Participant responses**

*Participant (combined patient and family/carer) responses to confidence associated Likert items*

| **Confidence statement** | **Strongly disagree** | **Disagree** | **Neutral** | **Agree** | **Strongly agree** |
| --- | --- | --- | --- | --- | --- |
| I am confident that I would notice if my/my family member’s medical condition suddenly or unexpectedly worsened | 0/132 (0%) | 1/132 (0.8%) | 5/132 (3.8%) | 64/132 (48.5%) | 62/132 (47%) |
| I would feel confident to tell a doctor or nurse of my concerns about a change in my/my family member’s medical condition | 0/133 (0%) | 0/133 (0%) | 1/133 (0.8%) | 49/133 (36.8%) | 83/133 (62.4%) |
| If the concerns I voiced were not addressed, I would ask to speak to a more senior nurse or doctor | 0/133 (0%) | 3/133 (2.3%) | 8/133 (6.0%) | 53/133 (39.8%) | 69/133 (51.9%) |
| If my concerns about a sudden or unexpected change in my/my family member’s medical condition were still not addressed, I would ask for the hospital’s RRT to be called | 3/131 (2.3%) | 10/131 (7.6%) | 23/131 (17.6%) | 44/131 (33.6%) | 51/131 (38.9%) |
| I do not feel confident to raise my concerns with doctors and nurses who do not usually look after me/my family member | 23/131 (17.6%) | 72/131 (55.0%) | 15/131 (11.5%) | 18/131 (13.7%) | 3/131 (2.3%) |
| I find it difficult to communicate changes in my/my family member’s medical condition to the doctors and nurses | 31/133 (23.3%) | 83/133 (62.4%) | 8/133 (6.0%) | 10/133 (7.5%) | 1/133 (0.8%) |
| I feel that I do not have sufficient medical knowledge to let the doctors and nurses know of any sudden or unexpected deterioration in my/my family member’s medical condition | 25/133 (18.8%) | 81/133 (60.9%) | 15/133 (11.3%) | 10/133 (7.5%) | 2/133 (1.5%) |

*Participant responses to barrier associated Likert items*

| **Barrier statement** | **Strongly disagree** | **Disagree** | **Neutral** | **Agree** | **Strongly agree** |
| --- | --- | --- | --- | --- | --- |
| Negatively scored |  |  |  |  |  |
| It is easier to raise concerns about my/my family member’s medical condition if the doctors and nurses ask me if I am concerned | 2/133 (1.5%) | 21/133 (15.8%) | 5/133 (3.8%) | 71/133 (53.4%) | 34/133 (25.6%) |
| It is easier to raise my concerns when I feel that the doctors and nurses value my opinion | 0/133 (0%) | 2/133 (1.5%) | 9/133 (6.8%) | 71/133 (53.4%) | 51/133 (38.3%) |
| In regard to my/my family member’s health, I know what is ‘normal’ for me/them | 0/133 (0%) | 1/133 (0.8%) | 8/133 (6.0%) | 79/133 (59.4%) | 45/133 (33.8%) |
| As a patient/family member, I have the right to tell staff if I have concerns about any sudden or unexpected deterioration in my/my family member’s medical condition | 0/133 (0%) | 0/133 (0%) | 0/133 (0%) | 41/133 (30.8%) | 92/133 (69.2%) |
| Positively scored |  |  |  |  |  |
| I am worried that if I express concerns about a sudden or unexpected change in my/my family member’s medical condition, I will upset or unnecessarily disturb the doctors and nurses | 41/133 (30.8%) | 78/133 (58.6%) | 6/133 (4.5%) | 8/133 (6.0%) | 0/133 (0%) |
| I am worried that if I express concerns about my/my family member’s medical condition, I will be negatively judged | 39/133 (29.3%) | 73/133 (54.9%) | 13/133 (9.8%) | 7/133 (5.3%) | 1/133 (0.8%) |
| I am worried that raising my concerns may have a negative impact upon my/my family member’s care | 37/133 (27.8%) | 74/133 (55.6%) | 10/133 (7.5%) | 12/133 (9.0%) | 0/133 (0%) |
| I am worried that if I express concerns about my/my family member’s medical condition, I will get the junior doctors and nurses into trouble | 44/133 (33.1%) | 69/133 (51.9%) | 12/133 (9.0%) | 7/133 (5.3%) | 1/133 (0.8%) |
| I feel that there is no need for me to raise concerns because the staff would be able to notice if there was a change in my/my family member’s condition | 17/133 (12.8%) | 57/133 (42.9%) | 19/133 (14.3%) | 30/133 (22.6%) | 10/133 (7.5%) |
| I feel that there is no need for me to raise concerns because the staff would know what is best for me/my family member | 16/133 (12.0%) | 61/133 (45.9%) | 24/133 (18.0%) | 25/133 (18.8%) | 7/133 (5.3%) |
| I rely on the doctors and nurses to notice if my/my family member’s medical condition is deteriorating | 6/133 (4.5%) | 38/133 (28.6%) | 15/133 (11.3%) | 51/133 (38.3%) | 23/133 (17.3%) |
