# Supplementary material for: A cross-sectional survey analysis of patient and family knowledge, confidence, and perceived barriers to reporting patient deterioration
Source: PLoS One. 2025 Mar 11;20(3):e0319546. doi: 10.1371/journal.pone.0319546 (PMC11896061; doi:10.1371/journal.pone.0319546)
Supplement: S4 File — (DOCX) [file pone.0319546.s004.docx]

**Supporting File S4: Unadjusted statistical analysis**

**Association between age and knowledge score**

In comparison to respondents in the <40 years age group, with all other factors remaining constant, participants aged 61-80 scored 3.1 points (12.4%) (95% CI 0.28, 5.96; p=0.032) higher and those >80 years. 3.4 points (13.6%) (95% CI -0.3, 7.16; p=0.071) higher.

**Confidence score patient versus family**

Scores were similar (95% CI -0.5, 1.91; p=0.25) and ranged from 23-35 (median = 28; IQR 27-31) for patients and 21-35 (median = 30; IQR 27-32) for family.

**Association between knowledge and confidence score**

There was a positive association between knowledge and confidence where a 5-point (20% increase) in the knowledge score was associated with a 0.91 point (3.3%) increase in confidence score (95% CI 0.34, 1.48; p=0.002).

**Association between knowledge and barrier score**

There was no significant association, between knowledge and barrier scores (p=0.54).

**Association between confidence and barrier score**

There was a negative association between confidence and barrier scores. For every 5 point (17.9%) increase in confidence, the barrier score decreased by 1.93 points (4.4%) (95% CI -2.99, -0.88; p<0.001).

**Association between barrier and confidence score**

A negative correlation (p<0.001) was identified between barrier and confidence scores. A 5-point (11.4%) increase in barrier score was associated with a 1.23 point (4.4%) decrease in confidence (95% CI -1.89, -0.56; p<0.001).

**Association between barrier and knowledge score**

There was no significant correlation between barriers and knowledge (p=0.54).

**Impact of barrier score on relationship between knowledge and confidence scores**

The positive association between knowledge and confidence scores differed significantly depending on the level of perceived barriers. This was strongest at the lowest barrier score of 12, where a 1-point (4%) increase in knowledge was associated with a 0.53-point (1.9%) increase in confidence (95% CI 0.2, 0.86; p=0.002), smaller at the mean barrier score of 23, where a 1-point (4%) increase in knowledge was associated with a 0.17-point (0.6%) increase in confidence (95% CI 0.06, 0.27; p=0.003), and no longer significant, but trending in the opposite direction, at the maximum barrier score of 36, where a 1-point (4%) increase in knowledge was associated with an average 0.27-point (1.0%) decrease in confidence (95% CI -0.66, 0.13, p=0.185).
